# The Lysin Exebacase Has a Low Propensity for Resistance Development in Staphylococcus aureus and Suppresses the Emergence of Resistance to Antistaphylococcal Antibiotics

**DOI:** 10.1128/spectrum.05261-22

**Published:** 2023-03-02

**Authors:** Jun Oh, Matthew Warner, Jane E. Ambler, Raymond Schuch

**Affiliations:** a ContraFect Corporation, Yonkers, New York, USA; University of California, San Diego

**Keywords:** lysin, direct lytic agent, serial passage, resistance, *Staphylococcus aureus*

## Abstract

Exebacase (CF-301) belongs to a novel class of protein-based antibacterial agents, called lysins (peptidoglycan hydrolases). Exebacase exhibits potent antistaphylococcal activity and is the first lysin to initiate clinical trials in the United States. To support clinical development, the potential for resistance development to exebacase was assessed over 28 days of serial daily subculture in the presence of increasing concentrations of the lysin performed in its reference broth medium. Exebacase MICs remained unchanged over serial subculture for three replicates each of methicillin-susceptible Staphylococcus aureus (MSSA) strain ATCC 29213 and methicillin-resistant S. aureus (MRSA) strain MW2. For comparator antibiotics also tested, oxacillin MICs increased by 32-fold with ATCC 29213 and daptomycin and vancomycin MICs increased by 16- and 8-fold, respectively, with MW2. Serial passage was also used to examine the capacity of exebacase to suppress selection for increased oxacillin, daptomycin, and vancomycin MICs when used together in combination, wherein daily exposures to increasing concentrations of antibiotic were performed over 28 days with the added presence of fixed sub-MIC amounts of exebacase. Exebacase suppressed increases in antibiotic MICs over this period. These findings are consistent with a low propensity for resistance to exebacase and an added benefit of reducing the potential for development of antibiotic resistance.

**IMPORTANCE** To guide development of an investigational new antibacterial drug, microbiological data are required to understand the potential for development of resistance to the drug in the target organism(s). Exebacase is a lysin (peptidoglycan hydrolase) that represents a novel antimicrobial modality based on degradation of the cell wall of Staphylococcus aureus. Exebacase resistance was examined here using an *in vitro* serial passage method that assesses the impact of daily exposures to increasing concentrations of exebacase over 28 days in medium approved for use in exebacase antimicrobial susceptibility testing (AST) by the Clinical and Laboratory Standards Institute (CLSI). No changes in susceptibility to exebacase were observed over the 28-day period for multiple replicates of two S. aureus strains, indicating a low propensity for resistance development. Interestingly, while high-level resistance to commonly used antistaphylococcal antibiotics was readily obtained using the same method, the added presence of exebacase acted to suppress antibiotic resistance development.

## INTRODUCTION

Resistance to traditional antibiotics coupled with a lack of new approved medical modalities to treat infections caused by drug- and multidrug-resistant pathogens poses a public health threat (1, 2). Altogether new chemotypes and innovative antimicrobial strategies are required to address this, as part of the next generation of therapeutic antimicrobial compounds for clinical use. One promising approach to killing antibiotic-resistant bacterial pathogens is based on direct lytic agents (DLAs), including lysins, which represent a new protein-based antimicrobial modality currently in clinical development (3).

Lysins are recombinantly produced cell wall-cleaving enzymes that elicit rapid peptidoglycan hydrolysis and resultant osmotic lysis (3–5). Exebacase (CF-301) is a lysin with potent bactericidal activity against Staphylococcus aureus and coagulase-negative staphylococci (CoNS) (6, 7). Exebacase can, furthermore, disrupt biofilms formed by a wide range of methicillin-susceptible and -resistant S. aureus (MSSA and MRSA, respectively) isolates as well as CoNS (8–12). The ability of exebacase to eradicate biofilm biomass and kill bacteria in biofilm has been demonstrated on a variety of surfaces, including catheters and other medical implants (8, 9).

In addition to the rapid bactericidal and antibiofilm activities of exebacase, other microbiological attributes include synergy with antistaphylococcal antibiotics, the absence of cross-resistance with antibiotics, and an extended postantibiotic effect (7, 13–15). A low propensity for the development of resistance to exebacase has also been reported, based on data generated using an *in vitro* serial passage methodology (7) utilizing cation-adjusted Mueller-Hinton broth (CAMHB) (16). Since these initial data were generated, the Clinical and Laboratory Standards Institute (CLSI) has approved the use of a modified medium that was developed for exebacase susceptibility testing together with quality control (QC) ranges to evaluate test performance with this medium (16, 17). This medium, comprised of CAMHB with horse serum (25% [vol/vol]) and 0.5 mM dl-dithiothreitol (pH 7.2 to 7.4), is referred to as CAMHB-HSD (16, 18). The use of 25% horse serum in CAMHB-HSD specifically enables consistent, reproducible MIC determinations across a range of clinical S. aureus isolates tested, including various resistance phenotypes, and under a variety of testing conditions (16).

To address the understanding of resistance to exebacase, studies are described here based on the serial (daily) subculture of S. aureus over 28 days in the presence of increasing concentrations of exebacase, performed in CAMHB-HSD (17, 18). Multiple replicates of two S. aureus strains (ATCC 29213 and MW2) demonstrated no change in exebacase susceptibility (evaluated by MIC determination) over a 28-day period of daily subculture. In contrast, high-level resistance to antibiotic comparators oxacillin, daptomycin, and vancomycin was generated. The serial passage method was also used to determine if the observed selection for antibiotic resistance could be suppressed by the addition of exebacase to the medium.

## RESULTS

### Initial MIC determination prior to serial passage studies.

Antibiotic and exebacase MIC values against MRSA strain MW2 and MSSA strain ATCC 29213 were determined as described in CLSI standard M07-A11 (19) as well as in CAMHB-HSD medium. The MIC data are presented in Table 1.

**TABLE 1:** MICs of exebacase and antibiotics against S. aureus strains

Bacterial strain	MIC (μg/mL) in medium:						
	CAHMB-HSD				CAMHB^*a*^		
	Exebacase	Daptomycin	Vancomycin	Oxacillin	Daptomycin	Vancomycin	Oxacillin
MW2 (MRSA)	0.5	0.5	1	ND^*b*^	0.5	1	ND
ATCC 29213 (MSSA)	0.5	ND	ND	0.5	ND	ND	0.5

a CAMHB was additionally supplemented with 50 μg/mL CaCl_2_ for the analysis of daptomycin and with 2% NaCl for the analysis of oxacillin.

b ND, not determined.

### Selection for decreased susceptibility to exebacase.

**FIG 1:**
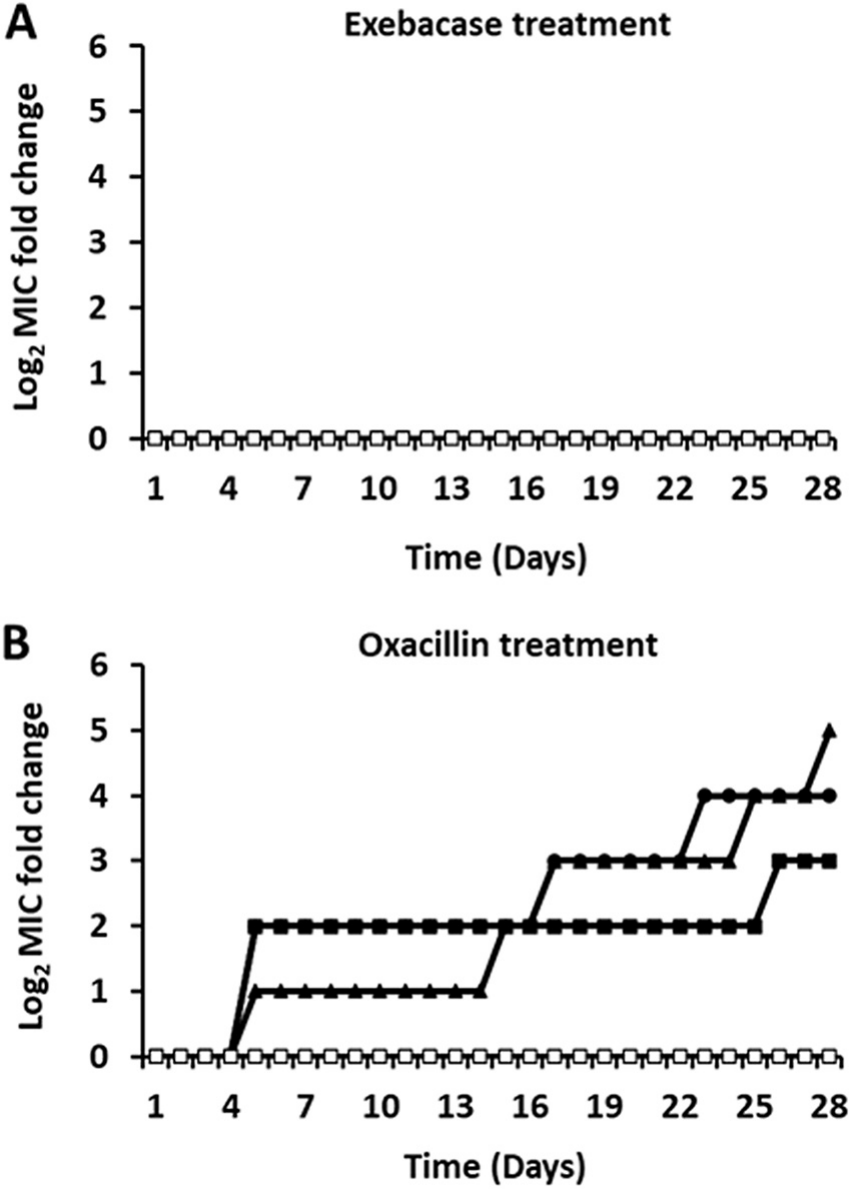
Serial passage of MSSA strain ATCC 29213 over 28 days with exebacase or oxacillin. (A) Log_2_ changes in exebacase MICs for three replicate cultures treated with exebacase and the untreated control in CAMHB-HSD. (B) Log_2_ changes in oxacillin MICs for three replicate cultures treated with oxacillin and the untreated passage control in CAMHB. Closed triangles, circles, and squares, three replicate treatments; open squares, untreated control.

**FIG 2:**
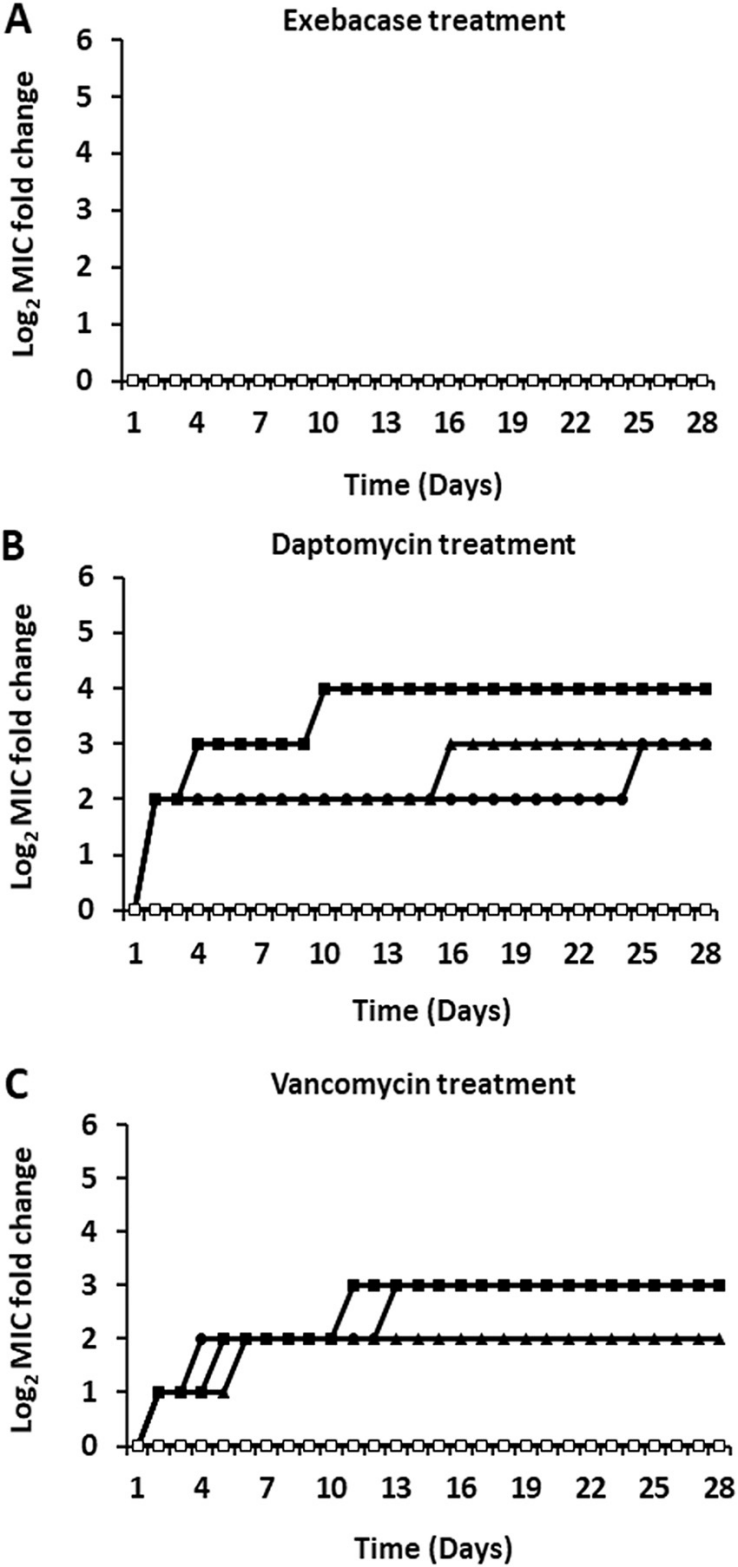
Serial passage of MRSA strain MW2 over 28 days with exebacase, daptomycin, or vancomycin. (A) Log_2_ changes in exebacase MICs for three replicate cultures treated with exebacase and the untreated control in CAMHB-HSD. (B) Log_2_ changes in daptomycin MICs for three replicate cultures treated with daptomycin and the untreated control in CAMHB (+CaCl_2_). (C) Log_2_ changes in vancomycin MICs for three replicate cultures treated with vancomycin and the untreated control in CAMHB. Closed triangles, circles, and squares, three replicate treatments; open squares, untreated control.

Methicillin-susceptible strain ATCC 29213 and MRSA strain MW2 were serially passaged with increasing concentrations of exebacase in CAMHB-HSD. After 28 days, no changes in exebacase MIC values were observed for three replicate passages of each S. aureus strain tested (Fig. 1A and Fig. 2A). In contrast, a stepwise resistance pattern was observed for oxacillin tested against ATCC 29213 in CAMHB (+NaCl), with MIC increases of up to 32-fold (from 0.5 μg/mL to 16 μg/mL) (Fig. 1B). A similar pattern was observed with daptomycin tested against MW2 in CAMHB (+CaCl_2_), with MIC increases of up to 16-fold (from 0.5 μg/mL to 8 μg/mL) (Fig. 2B). Vancomycin tested against MW2 in CAMHB likewise exhibited MIC increases of up to 8-fold (from 1 μg/mL to 8 μg/mL) (Fig. 2C).

### Suppression of oxacillin resistance by exebacase.

MSSA strain ATCC 29213 was serially passaged in CAMHB-HSD medium with increasing concentrations of oxacillin (with a starting range of 0.03 to 16 μg/mL) ± a sub-MIC of exebacase (1/16, 1/32, or 1/64 of the MIC). Prior to the study, each of these exebacase concentrations (corresponding to 0.03, 0.015, and 0.0075 μg/mL) was evaluated to have no influence on antibiotic MICs against either strain MW2 or strain ATCC 29213 (i.e., oxacillin, vancomycin, or daptomycin MICs were identical ± exebacase).

**FIG 3:**
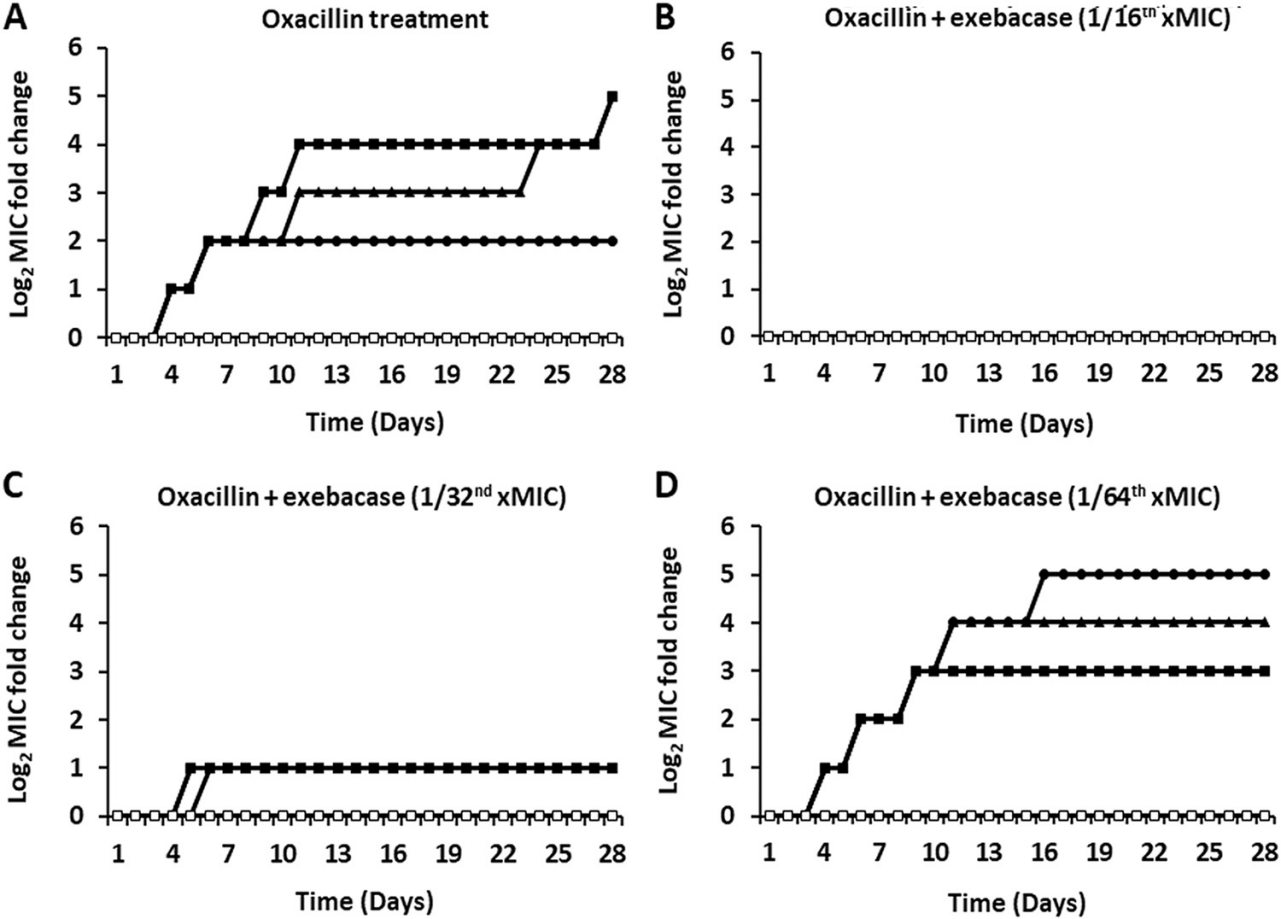
Oxacillin MICs through serial passage of MSSA strain ATCC 29213 over 28 days in CAMHB-HSD with oxacillin alone and in addition to sub-MIC exebacase. (A) Three replicate cultures treated with oxacillin alone and untreated control. (B) Three replicate cultures treated with oxacillin in addition to 1/16 of the MIC of exebacase (0.03 μg/mL). A control passaged only with sub-MIC exebacase is included. (C) Three replicate cultures treated with oxacillin in addition to 1/32 of the MIC of exebacase (0.015 μg/mL). A control passaged only with sub-MIC exebacase is included. (D) Three replicate cultures treated with oxacillin in addition to 1/64 of the MIC of exebacase (0.0075 μg/mL). A control passaged only with sub-MIC exebacase is included. Closed triangles, circles, and squares, three replicate treatments with oxacillin alone (A) or in addition to exebacase (B to D); open squares, untreated control (for oxacillin alone [A]) or exebacase alone (for combination passages [B to D]).

For oxacillin tested alone over 28 days (i.e., no exebacase present), oxacillin MICs increased by up to 32-fold, from 0.5 μg/mL to 16 μg/mL (Fig. 3A). The additional presence of the sub-MIC of exebacase suppressed this selection for higher oxacillin MICs, which remained unchanged at 0.5 μg/mL with exebacase at 1/16 of the MIC (0.03 μg/mL), (Fig. 3B) and increased by only up to 2-fold with exebacase at 1/32 of the MIC (0.015 μg/mL) (Fig. 3C). At the lowest concentration of exebacase tested, 1/64 of the MIC (0.0075 μg/mL), the suppression of oxacillin resistance was no longer observed (Fig. 3D).

### Suppression of daptomycin and vancomycin resistance by exebacase.

**FIG 4:**
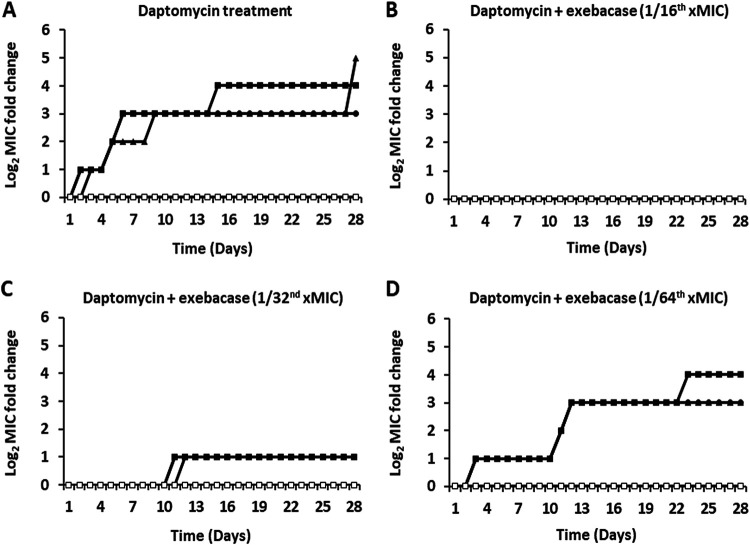
Daptomycin MICs through serial passage of MSSA strain ATCC 29213 over 28 days in CAMHB-HSD with daptomycin alone and in addition to sub-MIC exebacase. (A) Three replicate cultures treated with daptomycin alone and an untreated control. (B) Three replicate cultures treated with daptomycin in addition to 1/16 of the MIC of exebacase (0.03 μg/mL). A control passaged only with sub-MIC exebacase is included. (C) Three replicate cultures treated with daptomycin in addition to 1/32 of the MIC of exebacase (0.015 μg/mL). A control passaged only with sub-MIC exebacase is included. (D) Three replicate cultures treated with daptomycin in addition to 1/64 of the MIC of exebacase (0.0075 μg/mL). A control passaged only with sub-MIC exebacase is included. Closed triangles, circles, and squares, three replicate treatments with daptomycin alone (A) or in addition to exebacase (B to D); open squares, untreated control (for daptomycin alone [A]) or exebacase alone (for combination passages [B to D]).

**FIG 5:**
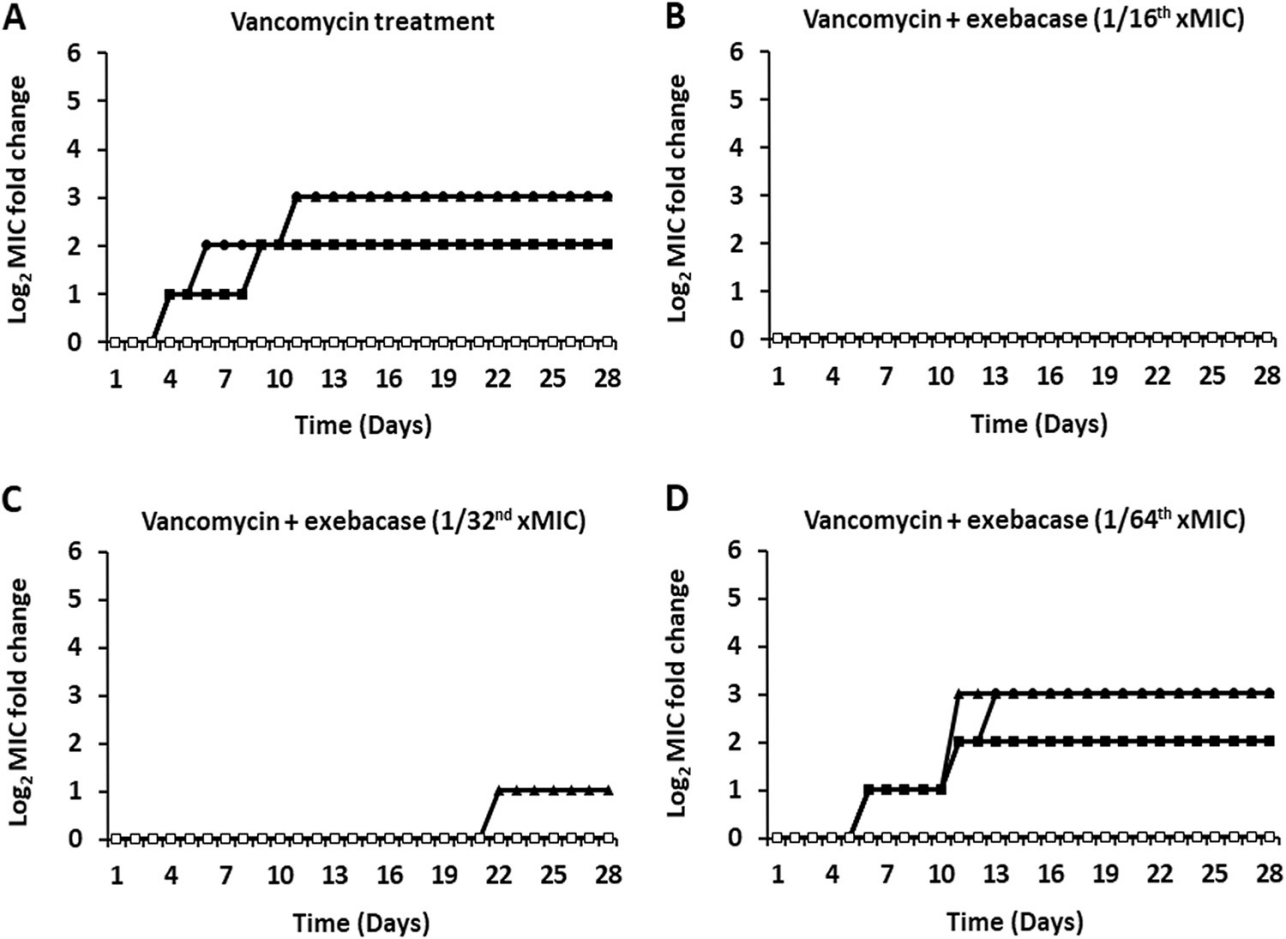
Vancomycin MICs through serial passage of MSSA strain ATCC 29213 over 28 days in CAMHB-HSD with vancomycin alone and in addition to sub-MIC exebacase. (A) Three replicate cultures treated with vancomycin alone and an untreated control. (B) Three replicate cultures treated with vancomycin in addition to 1/16 of the MIC of exebacase (0.03 μg/mL). A control passaged only with sub-MIC exebacase is included. (C) Three replicate cultures treated with vancomycin in addition to 1/32 of the MIC of exebacase (0.015 μg/mL). A control passaged only with sub-MIC exebacase is included. (D) Three replicate cultures treated with vancomycin in addition to 1/64 of the MIC of exebacase (0.0075 μg/mL). A control passaged only with sub-MIC exebacase is included. Closed triangles, circles, and squares, three replicate treatments with vancomycin alone (A) or in addition to exebacase (B to D); open squares, untreated control (for vancomycin alone [A]) or exebacase alone (for combination passages [B to D]).

MRSA strain MW2 was also serially passaged using CAMHB-HSD medium with increasing concentrations of daptomycin or vancomycin ± sub-MICs of exebacase (1/16, 1/32, or 1/64 of the MIC). For daptomycin and vancomycin tested alone over 28 days, daptomycin MICs increased up to 32-fold (Fig. 4A) and vancomycin MICs increased up to 8-fold (Fig. 5A). The presence of the sub-MIC of exebacase again suppressed selection for elevated antibiotic MICs, which remained unchanged, for daptomycin and vancomycin, with exebacase at 1/32 of the MIC (0.015 μg/mL) (Fig. 4B and Fig. 5B) and increased by only up to 2-fold with exebacase at 1/32 of the MIC (0.015 μg/mL) (Fig. 4C and Fig. 5C). At the lowest concentration of exebacase, 1/64 of the MIC (0.0075 μg/mL), the suppression of daptomycin and vancomycin resistance was no longer observed (Fig. 4D and Fig. 5D).

## DISCUSSION

Lysins are promising new protein-based antimicrobials comprised of bacteriolytic enzymes; several are currently under clinical development (20). These compounds are distinct from conventional antibiotics based on their unique physical and biochemical characteristics and rapid-acting mode of action—peptidoglycan hydrolysis causing destabilization of the cell wall and bacteriolysis. Consideration of these differences has given rise to a focused interest in the potential for emergence of resistance to lysin activity. The current understanding for the lysin class is of a low propensity for resistance development, based largely on *in vitro* studies in which serial exposures to escalating concentrations of multiple lysins were performed (21–28).

Microbiology development programs generate key translational data to guide clinical development of an investigational new drug. As exebacase is a first-in-class antibacterial agent, the potential for exebacase resistance development in S. aureus must be exhaustively studied to gain a comprehensive understanding. During early development, this was initially studied using an *in vitro* serial (daily) subculture method with MRSA strain MW2 incubated ± escalating concentrations of exebacase (7). Exebacase MICs increased by only 2- to 4-fold, while high-level resistance, with MIC increases of up to 128-fold, was observed for comparator antibiotics. It is important to note that these early data were generated using the CLSI broth microdilution reference method with CAMHB medium used to study traditional antibiotics, and not CAMHB-HSD medium, which was subsequently developed for exebacase susceptibility and approved by CLSI (18). Unlike CAMHB, CAMHB-HSD provides clear and reproducible MIC endpoint determinations for exebacase (16, 17).

Here, we report the low potential for development of resistance to exebacase using the serial subculture method with MRSA strain MW2 as well as MSSA strain ATCC 29213, using the appropriate medium, CAMHB-HSD, developed for exebacase susceptibility testing. Through 28 days of exposure, MICs of exebacase remained stable (i.e., no change was observed) for both methicillin-susceptible and -resistant strains; in contrast, high-level resistance was observed against the three commonly used antistaphylococcal antibiotics oxacillin, daptomycin, and vancomycin. These data further support the understanding that exebacase exhibits a low propensity for *in vitro* resistance development in S. aureus. The complete absence of exebacase MIC shifts in CAMHB-HSD, compared to the 2- to 4-fold increases previously observed in CAMHB, may be attributed to the presence of serum in CAMHB-HSD; certain serum proteins, specifically albumin and lysozyme (but not complement), have been shown to significantly enhance the antimicrobial effect of exebacase (29). The rapid nature of lysin activity against an abundant cell surface substrate (i.e., peptidoglycan) that is highly conserved among and required for the viability of susceptible species may, in part, explain the absence of exebacase resistance. Resistance to exebacase will be continually monitored throughout development.

Novel antimicrobials with reduced potential for resistance are attractive in the age of antibiotic resistance, in particular those used in addition to traditional antibiotics, as they not only appear to enhance the efficacy of each agent (i.e., synergy), but also appear to reduce the potential for antibiotic resistance to emerge. Considering the current treatment paradigms of exebacase (intravenous or local administration in addition to systemic antibiotic therapy), the impact of antimicrobial resistance was examined by serial passage either alone or in combination with commonly used antibiotics. The selection for increased MICs and high-level resistance to each of three antistaphylococcal antibiotics tested, oxacillin, daptomycin, and vancomycin, was effectively suppressed over a range of sub-MIC exebacase concentrations. These exebacase concentrations (corresponding to 0.03, 0.015, and 0.0075 μg/mL) were specifically chosen as having no influence on antibiotic MICs against either strain MW2 or strain ATCC 29213 (i.e., oxacillin, vancomycin, or daptomycin MICs were identical ± exebacase) in this work and in a previous synergy study (13). The concentrations were, however, in the range previously demonstrated to exert sub-MIC and postantibiotic effects on growth, ultrastructure, and the expression of virulence functions, possibly mediated through nonlethal effects on surface structure and metabolism (14). A more nuanced antimicrobial effect for exebacase, observed over a sub-MIC range, was suggested by this previous work and could also account for the suppression of antibiotic resistance observed in the current study.

Overall, these findings highlight the remarkable potential of exebacase (and possibly the lysin class) to serve as an adjunctive therapeutic strategy used in addition to conventional antibiotics to treat life-threatening bacterial infections, particularly those which result in substantial morbidity and mortality despite treatment with conventional antibiotics. Further studies to elucidate the mechanism(s) of sub-MIC effects on antibiotic resistance reported here are, in particular, warranted and may provide important mechanistic insights into previously reported observations (29) of the efficacy of exebacase at sub-MIC exposures when used in addition to conventional antibiotics in animal exposure target attainment models.

## MATERIALS AND METHODS

### Strains, reagents, and culture conditions.

The S. aureus MRSA strain MW2 (NRS123) was provided on request by the Network on Antimicrobial Resistance in Staphylococcus aureus (NARSA) for distribution by BEI Resources, NIAID, NIH (30). Staphylococcus aureus MSSA strain ATCC 29213 was obtained from the American Type Culture Collection (ATCC; Manassas, VA). Strains were propagated from frozen stocks by incubation for 24 h at 37°C on BBL Trypticase soy blood agar (TSAB) plates (Becton, Dickinson and Company, Sparks, MD). Exebacase was supplied at a concentration of 10.64 mg/mL in a liquid carrier solution containing proprietary components (ContraFect Corporation, Yonkers, NY). Daptomycin, vancomycin, and oxacillin were purchased from Sigma-Aldrich (St. Louis, MO), and stock solutions were prepared as described and stored at −20°C prior to use (18).

### Broth microdilution susceptibility testing.

Exebacase MICs were determined by broth microdilution using CAMHB-HSD medium (16, 18, 20), comprised of BBL Mueller-Hinton II (cation-adjusted) broth (catalog no. 212322, lot 1242967; Becton, Dickinson, Franklin Lakes, NJ) with horse serum (25% [vol/vol]) (catalog no. H1270, lot no. 21C414; Sigma-Aldrich, St. Louis, MO) and 0.5 mM dl-dithiothreitol (pH 7.2 to 7.4) (Sigma-Aldrich). As described in CLSI standard M07-A11 (20), daptomycin MICs were determined in CAMHB supplemented to 50 μg/mL with CaCl_2_, oxacillin MICs were determined in CAMHB with 2% NaCl, and vancomycin MICs were determined in CAMHB. In addition, oxacillin, daptomycin, and vancomycin MICs were generated against each strain using CAMHB-HSD. MIC panels were incubated for 18 to 20 h at 37°C.

### Serial passage resistance testing with single agents.

Single-agent resistance assays were performed in a manner similar to that described previously (7, 31, 32) using three independent replicate lineages of both S. aureus strain MW2 and S. aureus strain ATCC 29213, serially passaged over 28 days in the presence of escalating concentrations of exebacase over a 2-fold serial dilution range (0.03 to 16 μg/mL). The starting dilution range was chosen to bracket the initial exebacase MIC value of 0.5 μg/mL against strain MW2 and strain ATCC 29213, respectively. The inoculum for each daily passage was taken from the passage experiment of the previous day; specifically, the well containing the highest concentration of exebacase supporting growth on a particular day was used as the inoculum for the next day. Isolates generated at each step were subcultured twice on TSAB (in the absence of exebacase), and a confirmatory MIC analysis was performed. The log_2_ MIC fold change at each time point over the 28-day passage, compared to the starting MIC, was plotted. Control lineages were passaged in the absence of exebacase to evaluate the impact of serial subculture alone on exebacase MICs. Single-agent passages were also performed as described above for each antibiotic, with vancomycin passaged in CAMHB, oxacillin passaged in CAMHB with 2% NaCl, and daptomycin passaged in CAMHB supplemented to 50 μg/mL CaCl_2_. As above, all isolates generated at each step were subcultured twice on TSAB (in the absence of antibiotic), and a confirmatory MIC analysis was performed. All MIC shifts observed for antibiotics in serial passage were maintained after subsequent subculture in the absence of antibiotic and were, thus, examples of genotypic resistance.

### Serial passage resistance testing with antibiotics in addition to exebacase.

The suppressive effect of sub-MIC exebacase on selection for antibiotic resistance was examined in the 28-day serial subculture format. Three replicate cultures of MRSA strain MW2 were passaged with escalating (2-fold) concentrations of either daptomycin or vancomycin. Similarly, MSSA strain ATCC 29213 was passaged with oxacillin; antibiotic dilution ranges were chosen to bracket the MIC value at each daily time point. Unlike in the single-agent antibiotic passages above, the entire antibiotic dilution range was combined with a fixed sub-MIC amount of exebacase, either 1/16 of the MIC (0.03 μg/mL), 1/32 of the MIC (0.015 μg/mL), or 1/64 of the MIC (0.0075 μg/mL); thus, each row consisted of a range of antibiotic concentrations in addition to a constant concentration of exebacase. The antibiotic-plus-exebacase passage studies were all performed in CAMHB-HSD with the understanding that each of these antibiotics is active in this medium (Table 1) (13). Isolates generated at each step were subcultured twice on TSAB (in the absence of antibiotic or exebacase) and a confirmatory MIC analysis was performed. All MIC shifts observed for antibiotics in serial passage were maintained after subsequent subculture in the absence of antibiotic and were, thus, examples of genotypic resistance.
